# A Non-Pharmacological Paradigm Captures the Complexity in the Mechanism of Action of Poliprotect Against Gastroesophageal Reflux Disease and Dyspepsia

**DOI:** 10.3390/ijms26031181

**Published:** 2025-01-29

**Authors:** Sara Caterbi, Claudio Buttarini, Stefano Garetto, Isabelle Franco Moscardini, Stefano Ughetto, Angela Guerrini, Elena Panizzi, Cristiano Rumio, Laura Mattioli, Marina Perfumi, Anna Maidecchi, Andrea Cossu, Stanislas Bruley des Varannes, Jaroslaw Regula, Peter Malfertheiner, Claudia Sardi, Jacopo Lucci

**Affiliations:** 1 Bios-Therapy, Physiological Systems for Health S.p.A., Località Aboca 20, 52037 Sansepolcro, Italy; scaterbi@biostherapy.it (S.C.); cbuttarini@biostherapy.it (C.B.); sgaretto@biostherapy.it (S.G.); imoscardini@biostherapy.it (I.F.M.); sughetto@biostherapy.it (S.U.); aguerrini@biostherapy.it (A.G.); epanizzi@biostherapy.it (E.P.); csardi@biostherapy.it (C.S.); 2 Department of Pharmacology and Biomolecular Sciences, University of Milan, Via Trentacoste 2, 20134 Milan, Italy; cristiano.rumio@unimi.it; 3 Department of Experimental Medicine and Public Health, University of Camerino, Via Madonna delle Carceri 9, 62032 Camerino, Italy; lmattioli@biostherapy.it (L.M.); mperfumi@biostherapy.it (M.P.); 4 Aboca S.p.A, Società Agricola, Località Aboca 20, 52037 Sansepolcro, Italy; amaidecchi@aboca.it (A.M.); acossu@aboca.it (A.C.); 5 Department of Gastroenterology Hepatology and Clinical Oncology, Institut des Maladies de l’Appareil Digestif, Universitary Hospital, 44000 Nantes, France; sbdv54@gmail.com; 6 Department of Oncological Gastroenterology, Maria Sklodowska-Curie National Research Institute of Oncology, 00-001 Warsaw, Poland; jaroslaw.regula@nio.gov.pl; 7 Department of Gastroenterology, Hepatology and Clinical Oncology, Centre of Postgraduate Medical Education, 01-813 Warsaw, Poland; 8 LMU Klinikum Medizinische Klinik und Poliklinik II, Campus Großhadern, Marchioninistr. 15, 81377 München, Germany; peter.malfertheiner@med.ovgu.de; 9 Otto-von-Guericke Universität Magdeburg Klinik für Gastroenterologie, Hepatologie und Infektiologie, 39120 Magdeburg, Germany

**Keywords:** Poliprotect, NeoBianacid, gastroesophageal reflux disease, dyspepsia, biophysical properties, non-pharmacological mechanism, emerging properties, medical device, novel R&D paradigm

## Abstract

When the protective mechanisms of the gastroesophageal mucosa are overwhelmed by injurious factors, the structural and functional mucosal integrity is compromised, resulting in a wide spectrum of disorders. Poliprotect has recently been shown to be non-inferior to standard-dose omeprazole for the treatment of endoscopy-negative patients with heartburn and/or epigastric pain or burning. Here, we provide preclinical data describing the mechanism of action of the Poliprotect formulation, a 100% natural, biodegradable, and environmental friendly medical device according to EU 2017/745 and containing UVCB (unknown or variable composition, complex-reaction products, or biological materials) substances of botanical and mineral origin, according to the REACH and European Chemical Agency definitions. Different in vitro assays demonstrated the capability of Poliprotect to adhere to mucus-secreting gastric cells and concomitantly deliver a local barrier with buffering and antioxidant activity. In studies conducted in accordance with systems biology principles, we evaluated the effects of this barrier on human gastric cells exposed to acidic stress. Biological functions identified via Ingenuity Pathway Analysis highlighted the product’s ability to create a microenvironment that supports the mucosal structural and functional integrity, promotes healing, and restores a balanced mucosal inflammatory status. Additionally, transepithelial electrical resistance and an Ussing chamber showed the product’s capability of preserving the integrity of the gastric and esophageal epithelial barriers when exposed to an acid solution. Two in vivo models of erosive gastropathy further highlighted its topical protection against ethanol- and drug-induced mucosal injury. Overall, our findings sustain the feasibility of a paradigm shift in therapeutics R&D by depicting a very innovative and desirable mode of interaction with the human body based on the emerging biophysical, rather than the pharmacological properties of these therapeutic agents.

## 1. Introduction

The gastroesophageal mucosal barrier is a complex system involving physical, chemical, and biological defense mechanisms to meet the threat posed by either endogenous components, such as acid or destructive hydrolases, aggressors contained in the lumen, microorganisms, or exogenous noxious factors, including drugs (e.g., non-steroidal anti-inflammatory drugs (NSAIDs) and alcohol) [1,2,3,4,5,6]. These defense mechanisms are crucial to prevent upper gastrointestinal (GI) symptoms and damage, as well as to maintain structural and functional mucosal integrity.

Gastroesophageal reflux disease (GERD) and functional dyspepsia (FD) are common chronic upper GI disorders with an increasing prevalence worldwide (c. 14% and 8%, respectively) [7,8]. GERD is characterized by the regurgitation of stomach contents into the esophagus, causing troublesome symptoms—typically, heartburn and regurgitation—and possible complications. FD refers to a group of painful and painless symptoms with no identifiable organic cause (i.e., no lesions identified via endoscopy), accounting for the majority of patients with dyspepsia [9,10]. Similarly to dyspepsia, a minority of GERD patients show mucosal lesions at endoscopy, that are due to, and prove, pathological esophageal acid exposure, while around 70% have no mucosal abnormalities [11]. These patients are a heterogeneous group, including, as per the Rome IV consensus, non-erosive reflux disease (NERD: abnormal acid exposure at ambulatory reflux monitoring) and reflux hypersensitivity (RH) a GERD phenotype with typical reflux symptoms and positive reflux–symptom association despite normal acid exposure [12]. In a further analogy between these disorders, a multitude of factors other than acid, including hypersensitivity and/or the impaired integrity of the mucosa, play a pathogenic role, though their relative contribution and interdependence on a phenotype-wise base or in individual patients remains to be elucidated [13,14,15,16,17]. Lifestyle changes are helpful in the management of GERD and FD.

Acid-suppressant drugs, especially proton-pump inhibitors (PPIs), are highly effective therapeutic options for esophageal acid exposure and are commonly recommended as first-line therapy for both FD symptoms and bothersome heartburn [18,19,20], based on evidence of their efficacy [21,22,23]. However, PPIs are not devoid of adverse effects, including intestinal and lower respiratory tract infections and interactions with concurrent medications, requiring a carefully considered medical choice and vigilant oversight in special populations such as children [24,25,26,27,28]. Moreover, in keeping with the heterogeneity of phenotypes and the complex pathogenesis of these disorders, a sizeable proportion of GERD and FD patients do not respond to acid suppression; accordingly, there is a lack of consistency across controlled studies on the therapeutic benefits of acid secretion inhibitors in these patients [10,22,29,30,31,32]. A European Consensus Group stated that PPI therapy is an effective therapy for FD, whereas no other treatment approach reached consensus, yet there was no consensus that PPI therapy is the most appropriate initial therapy for FD [33]. As a matter of fact, there are currently no approved therapies for the treatment of FD in Europe. Prokinetics are another option for the pharmacological treatment of these patients, but with major limitations and drawbacks. Despite their theoretical benefits for treating GERD, there is no high-quality evidence for their use in GERD either as monotherapy or as an add-on treatment [34]. The evidence supporting the use of prokinetics in FD is rather poor, and no target subgroup can be defined based on the available evidence [33]. In addition, most prokinetics on the market carry some risk of neurological adverse effects and cardiac toxicity, including an increased likelihood of drug-induced, potentially fatal cardiac electrical abnormalities [35]. In this context, mucosal protectants, such as substance-based medical devices and alginates, provide a therapeutic option that has been shown, alone or as an add-on therapy, to meet the current management needs of GERD patients without esophagitis and, to a lesser extent, FD patients, as well as to address persistent reflux symptoms despite PPIs [34,36,37,38,39,40,41]. In principle, mucosal protective agents are developed to adhere to the gastroesophageal epithelium, reinforce the mucosal barrier, and protect the epithelium from acid and/or non-acid luminal components [36,37,38]. In a recent randomized, controlled, double-blind, double-dummy clinical trial with 275 endoscopy-negative outpatients, the Poliprotect formulation (Poliprotect_(F)_) proved non-inferior to standard-dose omeprazole for the relief of heartburn and epigastric pain/burning, indicating Poliprotect_(F)_ as a valid alternative treatment for PPIs in managing heartburn and painful clinical subtypes of FD in patients without mucosal lesions upon endoscopy [39]. In this article, we report on the complex mechanisms of action of Poliprotect_(F)_ from a series of experiments that show a synergistic beneficial effect on gastroesophageal mucosal integrity. Poliprotect_(F)_ is shown to adhere to the target mucosa and form a protective barrier, displaying several beneficial effects. The experiments included advanced methods of molecular and cellular biology, coupled with the application of bioinformatics tools to measure the overall biological outcomes of the devised activities, all capable of contributing simultaneously to the clinical performance of Poliprotect_(F)_.

## 2. Results

### 2.1. Assessment of Bioadhesive Properties of POLIPROTECT_(F)_

**Figure 1:**
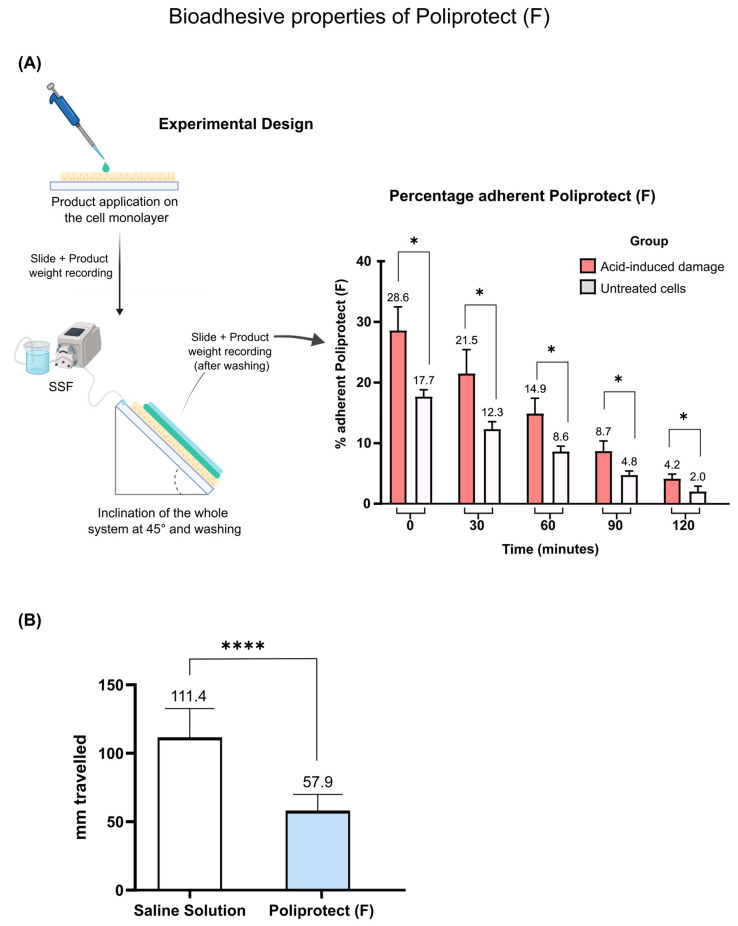
Bioadhesive properties of Poliprotect_(F)_: (**A**) Graphical representation of the percentage of Poliprotect_(F)_ remaining adhered on the human gastric mucus-secreting cell monolayer (NCI-N87) exposed (or not) to acid solution (pH 1) over time. SSF: simulated salivary fluid. Values are the mean ± SD; two-way ANOVA and Tukey’s post hoc test. (**B**) Graphical representation of the distance (mm) traveled by the device and saline solution on the surface of the plexiglass. Values are the mean ± SD of six separated experimental sessions; unpaired *t*-test. * *p*-value < 0.05; **** *p*-value < 0.0001.

Poliprotect_(F)_ is designed to act by forming a protective film covering the gastroesophageal mucosa. As shown in Figure 1A, Poliprotect_(F)_ was able to adhere to a human gastric mucus-secreting cell monolayer (NCI-N87): 17.7% at time 0, 12.3% at 30 min, 8.6% at 60 min, 4.8% at 90 min, and 2% at 120 min. Interestingly, the percentage of adhesion was higher in cells exposed to acid: 28.6% at time 0, 21.5% at 30 min, 14.9% at 60 min, 8.7% at 90 min, and 4.2% at 120 min. This result is remarkable, considering that acid is believed to be a primary trigger in the context of gastric mucosal damage. An assay conducted under abiotic conditions serves as a surrogate of the above-described one and further supports the non-pharmacological nature of this mechanism. The properties of the device were compared to a reference sample (saline solution) by measuring the distance traveled on an inclined plexiglass surface. The performance observed was 57.9 mm and 111.4 mm for Poliprotect_(F)_ and saline solution, respectively (Figure 1B).

### 2.2. Barrier Properties of Poliprotect_(F)_

**Figure 2:**
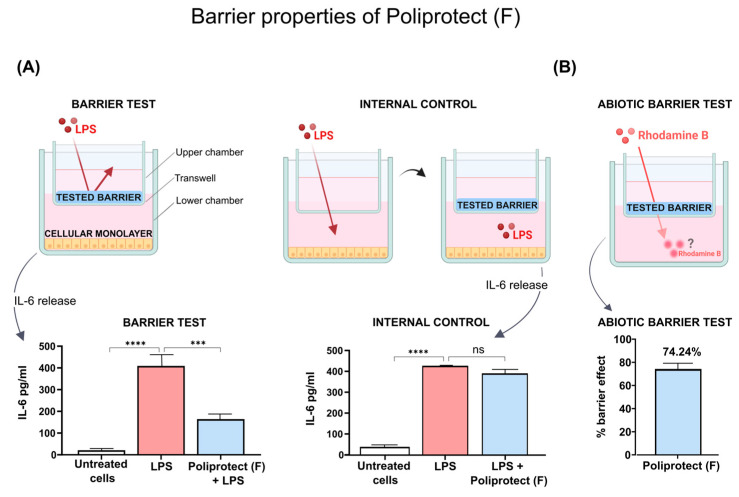
(**A**) Release of cytokine IL-6 in human fibroblasts in barrier test and internal control set-up. Values are the mean ± SD; ordinary one-way ANOVA and Dunnett’s post hoc test. (**B**) Barrier effect against the molecule dextran–Rhodamine B. *** *p*-value < 0.001; **** *p*-value < 0.0001. *ns*: not significant.

The inflammatory stimulus lipopolysaccharide (LPS) induced a significant increase in the release of interleukin 6 (IL-6) compared to untreated cells; see Figure 2A. The barrier test demonstrated that the release of IL-6 in response to stimulation with LPS was significantly reduced in human fibroblasts (HuDe cells) protected by Poliprotect_(F)_ compared to the positive control (Figure 2A). On the other hand, an evaluation of the effect of the potentially diffusing portion of Poliprotect_(F)_ within the receiving chamber, represented by the internal control (IC) experimental set-up, showed that there was no significant biological difference in IL-6 release between cells treated with the device and the positive control, indicating the absence of any meaningful interference with the synthesis of the cytokines (Figure 2A). Taken together, these data allow us to conclude that the observed anti-inflammatory activity depends on a mechanical effect of isolating cells from LPS, rather than on activities performed by interactors diffused from Poliprotect_(F)_. The impact of the treatments on cell viability in the internal control set-up and the barrier test was negligible, as detailed in Supplementary Figure S1. An assay conducted in abiotic conditions further strengthens the non-pharmacological nature of such a mechanism. The retention of Poliprotect_(F)_’s performance in these conditions also confirmed the physical/mechanical nature of this phenomenon. The application of Poliprotect_(F)_ to the surface of the transwell reduced the passage of the molecule dextran–Rhodamine B from the upper chamber to the lower chamber, highlighting its barrier effect (74.24%) (Figure 2B).

### 2.3. Radical Scavenging Activity

**Figure 3:**
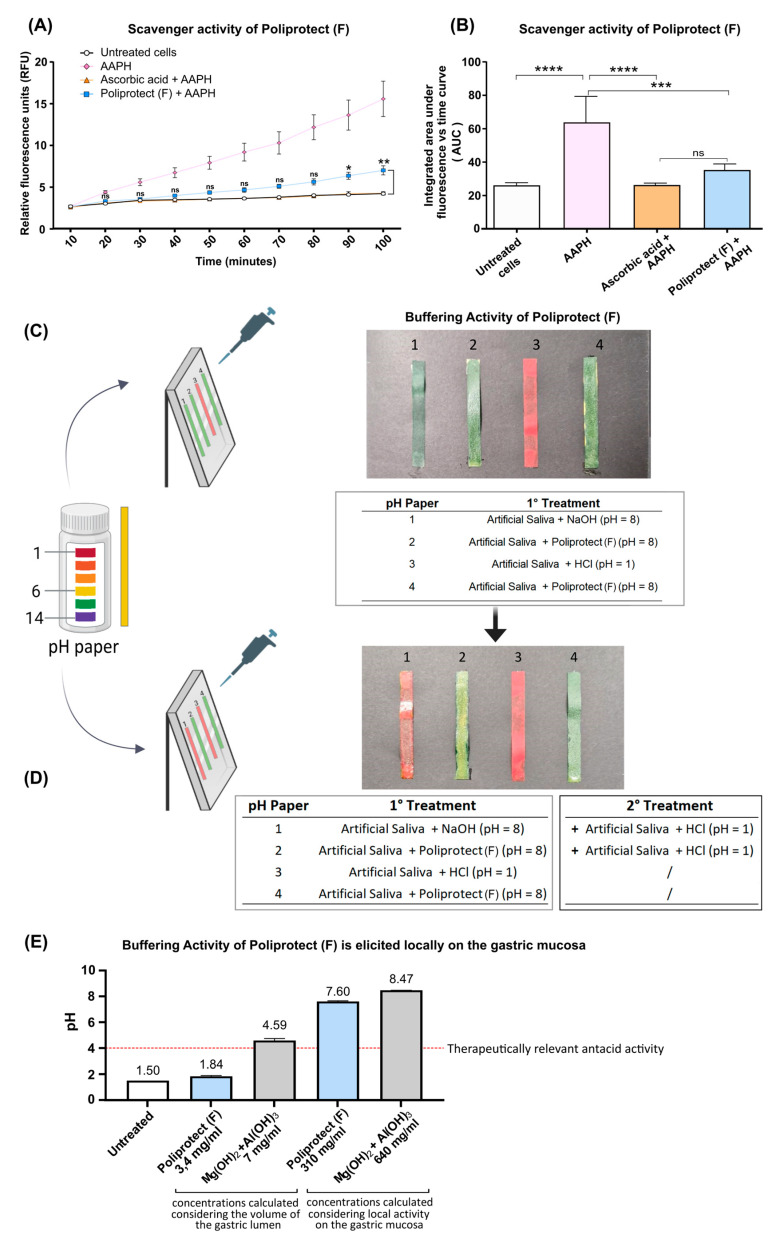
Radical scavenging activity and buffering activity of Poliprotect_(F)_: (**A**) Time-resolved curves of observed emitted fluorescence. The RFU measured was directly linked to the amount of ROS produced in human fibroblasts. The quantity of ROS detected in samples treated with AAPH + Poliprotect_(F)_ was compared with that measured in the AAPH + ascorbic acid sample. Values are the mean ± SD; two-way ANOVA with Dunnett’s post hoc test. (**B**) Integrated areas under the curves displayed in (**A**) were compared in order to quantify the total amount of fluorescence emitted over time. The quantity of ROS detected in samples treated with AAPH was compared with that measured in the remaining samples. Furthermore, the quantity of ROS detected in samples treated with AAPH + Poliprotect_(F)_ was compared with that measured in the ascorbic acid + AAPH sample. Values are the mean ± SD; one-way ANOVA and Sidak’s post hoc test were applied. For all tests, * *p*-value < 0.05, ** *p*-value < 0.01, *** *p*-value < 0.001, and **** *p*-value < 0.0001, *ns*: not significant. (**C**,**D**) Performance in terms of buffering barrier activity of Poliprotect_(F)_. (**E**) Buffering activity of Poliprotect_(F)_ is elicited locally on the gastric mucosa.

Treatment with 2,2-azobis(2-methylpropionamidine) dihydrochloride (AAPH) elicited a significant increase in the production of reactive oxygen species (ROS) in human fibroblasts (HuDe). From very early timepoints, Poliprotect_(F)_ counteracted the accumulation of free oxygen radicals, arguing in favor of the presence of radical scavenging activity. No significant difference in ROS production was observed in cells treated with AAPH + Poliprotect_(F)_ and AAPH + ascorbic acid (Figure 3A,B).

### 2.4. Buffering Activity and Its Localization

Consistent with its acidic nature, a pH strip covered with Hanks’s Balanced Salt Solution (HBSS) at pH 1 turned red (Figure 3C). The strip with the Poliprotect_(F)_ layer turned green, indicating an alkaline pH value of around 8. Similarly, the strip covered with the basic solution at pH 8 turned red when exposed to HBSS at pH 1. Importantly, though, when the Poliprotect_(F)_ layer was challenged by adding the same pH 1 HBSS, the pH value remained stable, highlighting the ability of the device to create a protective effect that does not hinge exclusively on the capability to buffer, arguing in favor of the constitution of a buffering barrier (Figure 3D).

To determine the localization and therapeutic relevance of Poliprotect_(F)_’s buffering activity, we studied its capability to buffer an acid solution at pH values typical of the gastric levels during digestion [42,43], across a range of concentrations that would signify either a local or a luminal mechanism of action. The commercial product based on magnesium hydroxide 400 mg + aluminum oxide 400 mg proved to be effective in the scenario mimicking an activity elicited in the gastric lumen: the pH increased to >4, and it was also obviously maintained when tested in the scenario representing an activity elicited locally on the mucosa, which implies the use of an even higher test item concentration. Conversely, the buffering performance of Poliprotect_(F)_ can be considered therapeutically relevant only when a concentration that implies that local activity on the gastric mucosa is studied (Figure 3E).

### 2.5. Effects of Treatment with Poliprotect_(F)_ on Human Gastric Epithelial Cells

**Figure 4:**
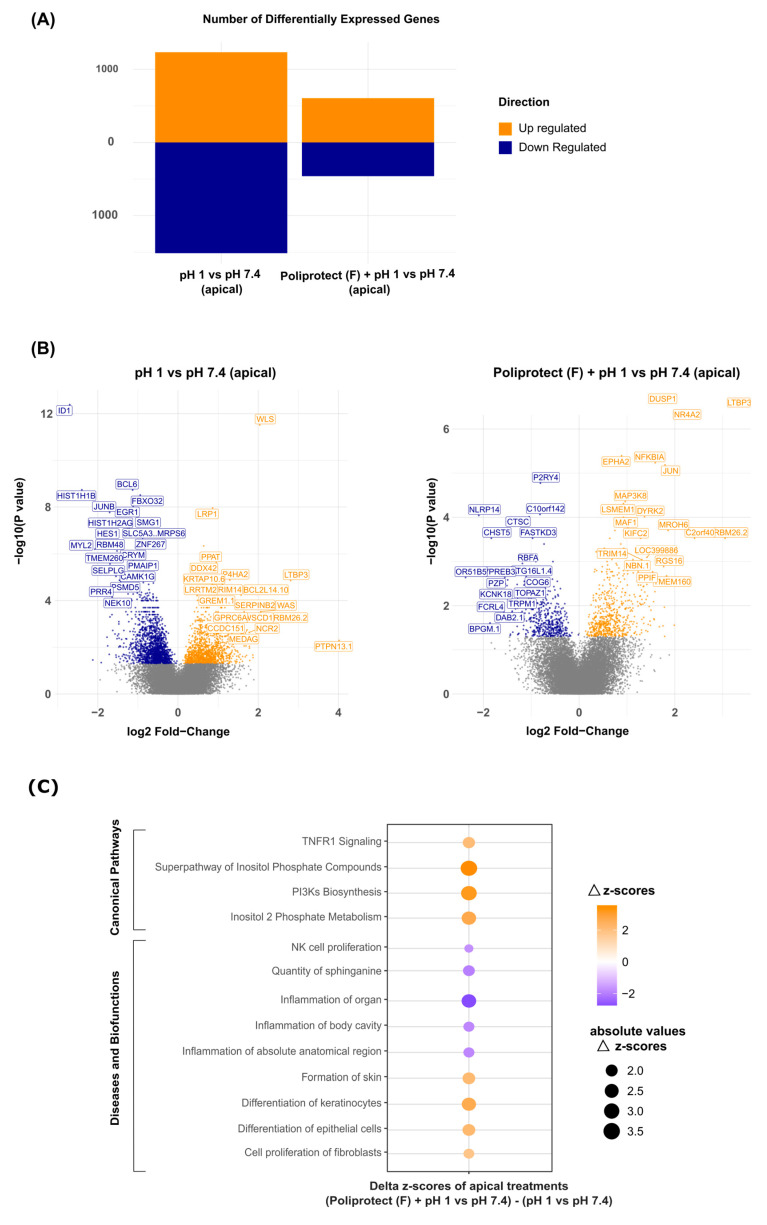
(**A**) Number of differentially expressed genes after acid-induced damage in the absence and presence of Poliprotect_(F)_ apical treatment (experimental design in Supplementary Figure S2) in human gastric epithelial cells. The gene expression modulation is determined by a color code: blue (downregulation) and orange (upregulation). (**B**) Volcano plot displaying the differentially expressed genes in the same conditions described in (**A**). Orange dots represent significantly upregulated genes, while blue dots represent downregulated genes. Grey dots represent not significant genes. The respective gene symbols for the most significant transcripts are displayed (higher fold-change values and lower *p*-values). (**C**) Dot plot representing the differences in the modulation of canonical pathways and diseases and biofunctions between Poliprotect_(F)_ treatment at pH 1 and physiological pH 7.4, as well as the acidic environment (pH 1) versus physiological pH (7.4) (the delta of the z-scores obtained for each comparison). The color code follows the same rule described above, and the size of the dots represents the absolute value of the differences in the z-scores.

**Figure 5:**
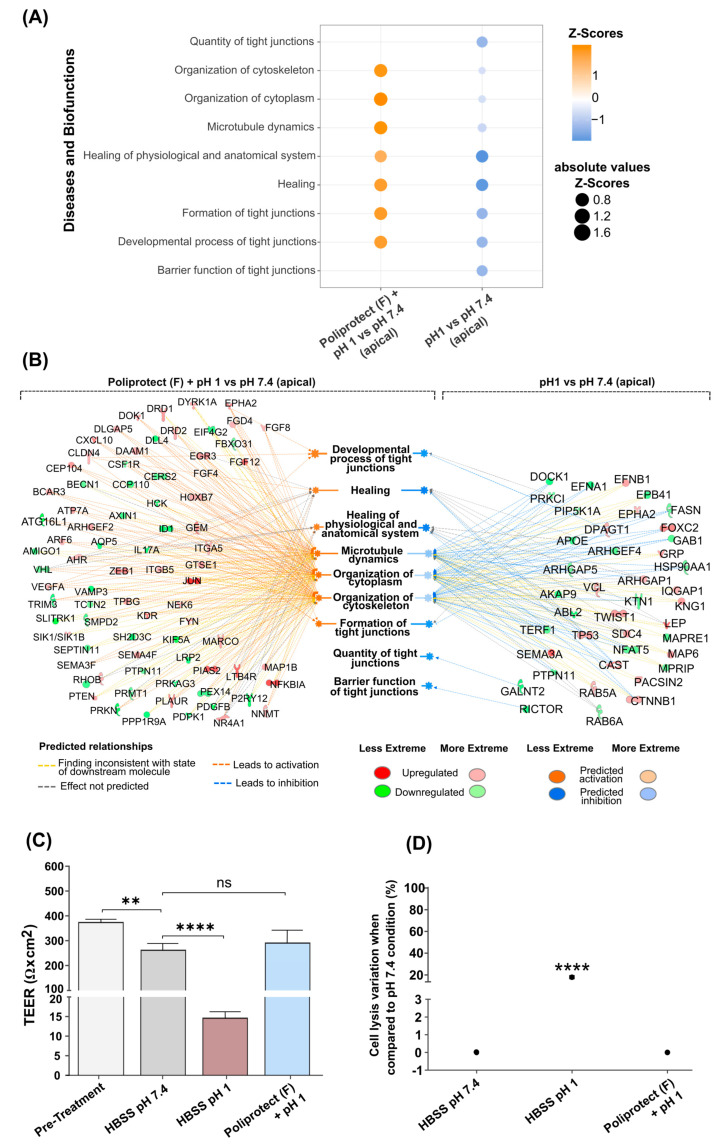
(**A**) Dot plot displaying inquired biofunctions; z-scores were calculated based on the reference values of color codes provided via IPA (Supplementary Figure S4). Positive z-scores are represented in orange, while negative values are represented in blue. The size of the dots represents the absolute value of the z-score. (**B**) IPA network representing the genes involved in the modulation of the inquired diseases and biofunctions. The network displays the relationship for both comparisons: (i) apical acidic solution (HBSS, pH 1) versus the physiological control (pH 7.4), and (ii) previous Poliprotect_(F)_ treatment at pH 1 versus the physiological control. Genes are colored according to the findings in the transcriptomics analysis: red represents upregulated genes, while green represents downregulated ones Diseases and biofunctions predicted to be up- and downregulated are displayed in orange and blue, respectively (Path Designer Shapes in Supplementary Figure S5). On the opposite side, orange lines represent relationships that lead to the activation of the inquired diseases and biofunctions, lines in blue represent those that lead to inhibition, yellow lines represent findings that are inconsistent with the trend of the modulation of the biofunctions obtained, and gray lines represent those in which the effect was not predicted. (**C**) The transepithelial electrical resistance of human gastric epithelial cells (NCI-N87) exposed to HBSS at pH 7.4 or pH 1. Values are the mean ± SD; one-way ANOVA with Dunnett’s post hoc test. ** *p*-value < 0.01; **** *p*-value < 0.0001. (**D**) Graphical representation of the viability of the human gastric epithelial cell monolayer, assessed by measuring the release of the enzyme adenylate kinase (AK) present in the solution where cell membrane integrity was compromised. Values are the mean ± SD; one-way ANOVA with Dunnett’s post hoc test. **** *p*-value < 0.0001, *ns*: not significant.

Human gastric epithelial cells (NCI-N87) that had been treated as reported in the Section 4 were tested to identify gene expression regulation on a whole-genome scale. The software Ingenuity Pathway Analysis (IPA) was then used to extract biological meaningfulness from the transcriptomics data. The gene expression modulation was determined by a color code: blue (downmodulation) and orange (upmodulation). According to the significance of the differential expression analysis performed with the limma approach (*p*-value < 0.05), the comparison of the insult (pH 1) versus the physiological baseline (pH 7.4) led to 1517 downregulated and 1235 upregulated genes, while the same analysis with previously Poliprotect_(F)_-treated cells resulted in 463 downregulated and 607 upregulated genes (Figure 4A). Volcano plots, using the same color code, display the transcripts that exhibited differential expression after acid-induced damage alone or with previous apical Poliprotect_(F)_ treatment. The respective gene symbols were added for the most significant transcripts (characterized by higher fold-change values and lower *p*-values) (Figure 4B). IPA-associated trends with canonical pathways and biofunction regulation in different experimental conditions are shown using a color code: orange (upmodulation) and blue (downmodulation) (Figure 4C). The analysis was represented as the difference between “Poliprotect_(F)_ apical treatment versus physiological pH (7.4)” for “acidic environment (pH 1) versus physiological pH (7.4)”. Consistent with our expectations, the results show that the disruption of tight junctions and the loss of epithelial barrier function caused by acid treatment increase the permeability of the epithelium to injurious factors, leading to inflammation and disorganization of cellular junctions (Figure 5A). The perturbed status of the homeostatic condition induced via the acidic environment is highlighted by the downmodulation of biofunctions related to tight junctions and cytoskeletal organization. These events are associated with an alteration of the physiological mechanisms of healing (Figure 5A). The layer formed by Poliprotect_(F)_ is able to counteract the inflammatory response elicited via the acid treatment, preventing the onset or perpetuation of damage. Moreover, the results highlight the capability of Poliprotect_(F)_ to protect the stability of tight junctions and of the cytoskeleton, thereby preserving their functional structure, which is crucial to ensuring the physiological barrier function. The data also reveal the ability of Poliprotect_(F)_ to preserve cell renewal, a phenomenon that is crucial to sustaining the mucosa’s structural integrity, function, and healing. The gene expression profile was further investigated in order to explore the impact of cell treatment on additional biofunctions of interest in order to provide further details concerning the protection offered via the device, highlighting the ability of Poliprotect_(F)_ to sustain the physiological regenerative processes of the damaged epithelium. An IPA network representation (Figure 5B) of acid-induced damage and Poliprotect_(F)_ apical treatment further highlighted the benefits of mucosal protection offered via Poliprotect_(F)_.

The gene expression profile observed when Poliprotect_(F)_ was applied to the basolateral side (experimental design, Supplementary Figure S2) was also analyzed (Supplementary Figure S3). The results show the inability of the device to exert any protective activity when dissolved in solution at a concentration mimicking the worst-case scenario, in which 100% of the product is systemically absorbed.

Consistent with the gene expression data, the measurement of TEER was used to monitor the permeability [44] of the tested epithelial monolayer, confirming that Poliprotect_(F)_’s protective activity is useful to preserve the integrity of the epithelium against a relevant acidic environment (Figure 5C). Maintenance of the stability and electrical resistance of an epithelium is critical for essential physiological processes; therefore, significant changes in TEER represent an early expression of cell damage. The TEER values measured before treatment conditions confirm the ability of human gastric epithelial cells (NCI-N87) to form a tight monolayer [45]. The damage control (experimental design in Supplementary Figure S2), represented by the acid solution (pH 1), induced a significant decrease in TEER values compared to HBSS pH 7.4, indicating a loss of monolayer integrity. Conversely, no effects on TEER were observed when Poliprotect_(F)_ was applied to NCI-N87 cell monolayers compared to HBSS at pH 7.4. Concerning cell viability, only exposure of NCI-N87 cells to HBSS (pH 1) induced a significant reduction in viability compared to the other treatment conditions, signifying that the barrier generated via the device can effectively protect against damage (Figure 5D).

### 2.6. Preservation of the Permeability of Gastric and Esophageal Mucosae

**Figure 6:**
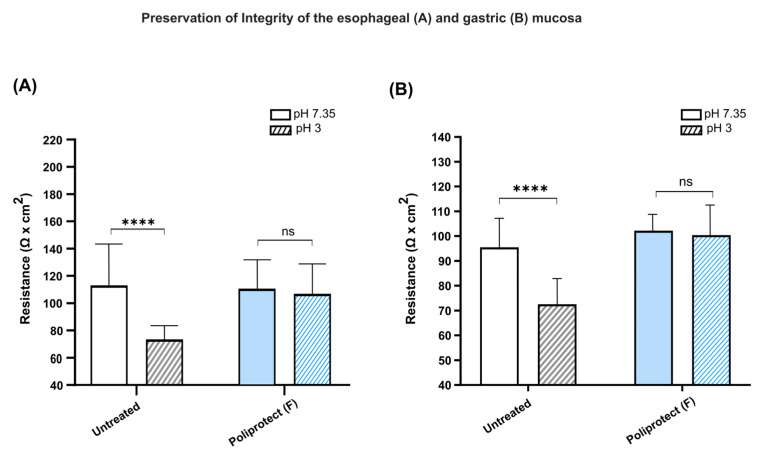
Preservation of the permeability of the gastric and esophageal mucosae: (**A**) Transepithelial electrical resistance of murine esophageal mucosae exposed to Krebs buffer at pH 7.35 or pH 3. (**B**) Transepithelial electrical resistance of murine gastric mucosae exposed to Krebs buffer at pH 7.35 or pH 3. For all tests, values are the mean ± SD (*n* = 5); unpaired *t*-test. **** *p*-value < 0.0001, *ns*: not significant.

Consistent with the data obtained via the transcriptomics approach and on cell monolayers, the protective activity of Poliprotect_(F)_ in terms of the preservation of the integrity of the epithelial surface was further confirmed in ex vivo studies. The integrity of the tissues depends on the mechanical cohesion between cells, and tight junctions are mainly responsible for this cohesion. Any significant changes in TEER mirror early injury to the cells and to the cohesive forces between them [1,2,3]. In these experiments, we first measured the TEER of esophageal mucosae from mice treated in vivo with Poliprotect_(F)_ or with a control solution (Figure 6A). To induce damage to the esophageal mucosa, the buffer solution in the Ussing system was replaced with an acid solution at pH 3. The resistance value is proportional to membrane integrity, so the greater the variation in resistance, the greater the tissue damage found. Acid insult reduces the integrity of the gastric membranes. The untreated control showed a significant change in resistance (113 vs. 73.49 ohm × cm^2^). Conversely, the reduction in electric resistance upon exposure to acid was significantly smaller in gastric tissue protected by the device compared with untreated tissue, indicating efficient protection (110.8 vs. 106.82 ohm × cm^2^). Equivalent experiments were then performed on gastric mucosae (Figure 6B). The results showed that acid attack reduces the integrity of the gastric membranes. The untreated control showed a significant change in resistance (95.50 vs. 72.53 ohm × cm^2^). Conversely, the passage from pH 7.35 to pH 3 did not cause a change in resistance on the mucosa treated with the product (102.14 vs. 100.42 ohm × cm^2^), indicating efficient protection.

### 2.7. Lack of Direct Anti-Inflammatory Activity in a Human Macrophage Model

**Figure 7:**
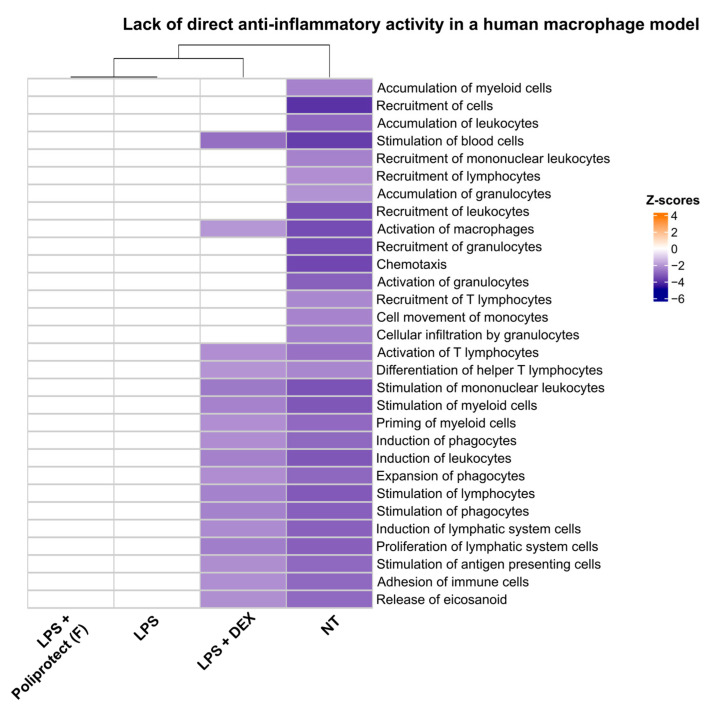
Lack of direct anti-inflammatory activity in a human macrophage model (U937 model): Heatmap representation of biofunctions’ modulation observed in response to different treatment conditions. Cells treated with Poliprotect_(F)_ and then inflamed with LPS could not be distinguished from cells inflamed with LPS alone. On the other hand, cells treated with the anti-inflammatory drug dexamethasone and then inflamed with LPS were characterized by a pattern of activation/inhibition of biofunctions similar to that of untreated (NT), non-inflamed cells.

To provide further evidence that the anti-inflammatory activity of Poliprotect_(F)_ depends on its ability to form a barrier locally in the GI tract and not on any activity exerted directly on immune cells as a consequence of its absorption, the inability of Poliprotect_(F)_ to counter inflammation evoked via LPS in a U937 cell model was verified at a concentration mimicking the worst-case scenario, in which 100% of the device is systemically distributed. A heatmap representation of biofunctions’ modulation, calculated using IPA and observed in response to different treatment conditions in U937 cells, is shown in Figure 7. Several different biological functions clearly defining an enhanced inflammatory state were strongly activated upon treatment with LPS compared to untreated cells. The addition of dexamethasone effectively counteracted the establishment of an inflammatory state, and this was unsurprisingly mirrored by the repression of biological functions activated via LPS alone. In contrast, cells treated with Poliprotect_(F)_ and then inflamed with LPS failed to counteract the establishment of an inflammatory state and were still described by a pattern of activation/repression of relevant biological functions, rendering them indistinguishable from cells that were fully inflamed when treated with LPS alone. These observations were confirmed by the results of a clustering analysis performed on the patterns of activation/repression of several biological functions generated in response to different treatment conditions. The dendrogram at the top of the heatmap displays the degree of similarity between different patterns. Cells treated with LPS alone or with the product and subsequently with LPS were defined by the same pattern of activation/repression of biological functions relevant to a well-established inflammatory state. Cells treated with dexamethasone and subsequently inflamed with LPS showed a different pattern of activation/repression of biological functions, signifying anti-inflammatory activity. Thus, the results further show that Poliprotect_(F)_ protective activity is exerted locally in the GI tract.

### 2.8. Protective Activity of Poliprotect_(F)_ In Vivo in an Animal Model of Erosive Gastropathy

**Figure 8:**
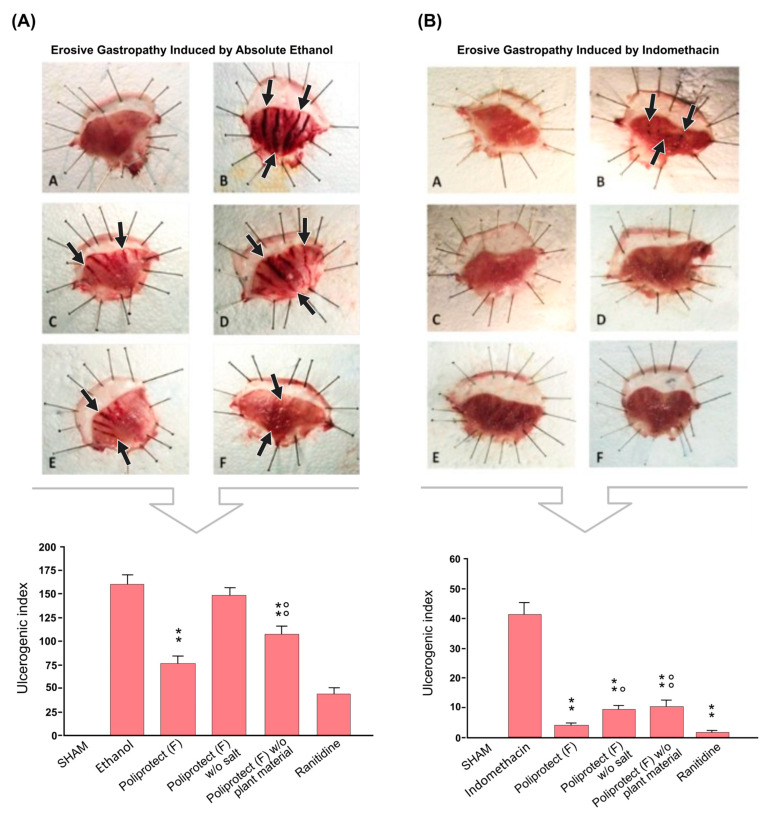
Protective activity of Poliprotect_(F)_ in vivo: Erosive gastropathy induced via (**A**) absolute ethanol and (**B**) indomethacin. Images of prepared morphological samples opened and extended to evaluate the presence of ulcers (indicated by black arrows) on the gastric mucosa after ethanol administration. Effects of treatments on ethanol- or indomethacin-induced gastric lesions are expressed as an ulcerogenic index. (**A**): SHAM (rats without lesions, treated with vehicle). (**B**): Ethanol or indomethacin (rats treated with ethanol or indomethacin). (**C**): Poliprotect_(F)_. (**D**): Poliprotect_(F)_ without (w/o) salt. (**E**): Poliprotect_(F)_ without (w/o) plant material. (**F**): Ranitidine. Values are the mean ± s.e.m (*n* = 6/12). ** *p*-value < 0.01 versus ethanol; °° *p*-value < 0.01 versus Poliprotect_(F)_; ** *p*-value < 0.01 versus indomethacin; ° *p*-value < 0.05 and °° *p*-value < 0.01 versus Poliprotect_(F)_.

The ethanol-treated group showed characteristic mucosal lesions. A macroscopic analysis of the stomach showed how treatment with ethanol in rats significantly increased the formation of gastric lesions resembling ulcers compared to untreated animals (SHAM). The lesions were very evident in the stomachs of rats treated only with alcohol, with massive damage to the gastric wall, the formation of extended ulcerated areas, and the presence of edema distributed along the entire wall of the stomach (Figure 8A(B)). Pre-treatment with Poliprotect_(F)_ effectively protected the mucosal layer, as demonstrated by the ~50% reduction in gastric lesions caused by ethanol (75.67 ± 8.746 vs. 159.75 ± 10.773) (Figure 8A(C)). A reduction in the ulcerogenic index equal to approximately 30% was also observed following treatment with Poliprotect_(F)_ without plant material (106.67 ± 8.862) (Figure 8A(E)). This effect was significantly less than that observed with Poliprotect_(F)_. No significant reduction in the number of ulcers was observed after treatment with Poliprotect_(F)_ without salt (147.58 ± 9.408) (Figure 8A(D)). Treatment with ranitidine indeed significantly reduced the formation of gastric ulcers (42.83 ± 7.650) (Figure 8A(F)). A macroscopic analysis of the stomach showed that treatment with indomethacin in rats significantly increased the formation of gastric lesions resembling ulcers compared to untreated animals (SHAM). Treatment with indomethacin caused the formation of necrotic areas and punctiform edema, which were distributed in different regions of the stomach (Figure 8B(B)). Pre-treatment with Poliprotect_(F)_ effectively protected the mucosal layer, as demonstrated by the >90% reduction in gastric lesions caused by indomethacin (3.667 ± 0.772 vs. 41.083 ± 3.803) (Figure 8B(C)). A reduction in the ulcerogenic index equal to approximately 70% was also observed following treatment with Poliprotect_(F)_ without plant material (10.167 ± 2.092) (Figure 8B(E)) and Poliprotect_(F)_ without salt (9.167 ± 1.043) (Figure 8B(D)). Treatment with ranitidine significantly reduced the formation of gastric ulcers (1.417 ± 0.417) (Figure 8B(F)).

## 3. Discussion

Several pharmacological and non-pharmacological therapies are available to treat GERD and FD [31,34,46,47]. The efficacy and safety of any prescribed treatment are critically dependent on several factors, such as ensuring that the treatment is appropriate for the specific clinical condition, the choice of using the treatment as a main or add-on therapy, careful consideration of the benefit-to-risk balance, and possible causes of symptom persistence despite treatment [31,34,46]. In such complex disorders, the development of solutions with broad, rather than pinpointed, extremely targeted actions and effects, duly verified for clinical efficacy and safety, may validly expand the current therapeutic pharmacological scenario [34].

This may be the case with UVCB substances; the complexity of their formulation translates into a great degree of complexity in their interactions with the human body, which fails to be recapitulated through the “lock and key” model associated with pharmacological activity.

An appreciation of such distinctions, such as the ability of therapeutic options to interact with a biological system via the establishment of a network of a variety of biophysical interactions, rather than a very well-defined punctual interaction with a single receptor, was possible here due to the use of advanced omics techniques and innovative cell-based assays, preventing the need to resort to using the (in this case) inadequate conceptual tools typical of the study of isolated molecules exerting pharmacological effects, such as those describable via the “lock and key” model.

**Figure 9:**
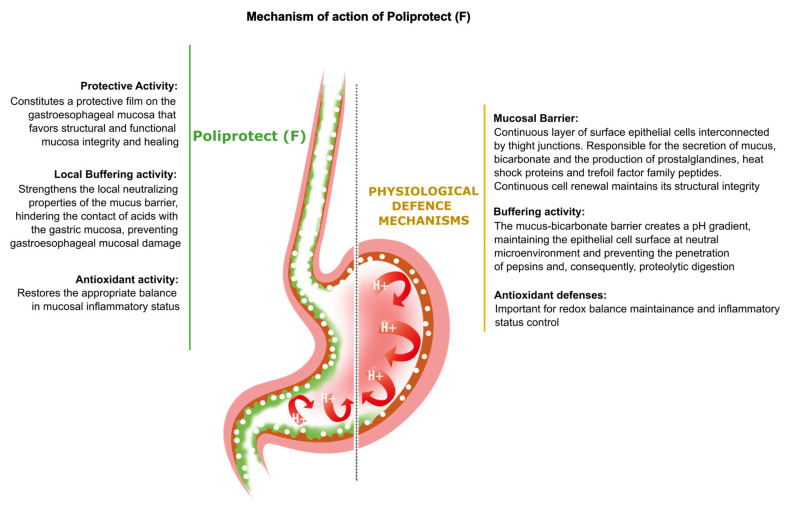
Schematic cartoon of the mechanism of action of Poliprotect_(F)_.

In the present study, we offer a novel paradigm for approaching the above-described scenario, reporting the benefits of mucosal protection offered by Poliprotect_(F)_, which acts as a single entity acquiring emerging properties. Such properties are typical of the complex matrix representing the final device, and they are different from those of its individual components when they are studied in isolation [48,49,50]. All components of the product actively participate in determining the mechanism of action and, therefore, the efficacy profile. Such phenomena can impact not only quantitative aspects of the behavior of single components in the mixture but also qualitative ones, such as their dynamic behavior per se. It is thus intended that the properties that are known to be associated with individual components of the mixture are not automatically transferred to the final mixture in an additive fashion, but as a result of the establishment of functional and structural interactions between the different molecular components, new properties can emerge that are associated specifically with the final mixture in a process known as the establishment of a matrix effect. In the case of Poliprotect_(F)_, as a non-exhaustive example, the bulk of our data demonstrate that an anti-inflammatory outcome becomes associated exclusively with a protective buffering barrier with antioxidant properties, rather than phenomena mediated via pinpointed pharmacological mechanisms of action based on a “lock and key” interaction of components of the mixture with specific receptors. As demonstrated here, such a paradigm finally grants access to a novel route that allows for the possibility of testing complex preparations in their entirety, rather than assuming that their behavior would result from the sum of those known for their components. Using a multidisciplinary combination of in vitro, ex vivo, in vivo, and in silico studies, we focused on the characterization of Poliprotect_(F)_’s emerging properties as a product containing UVCB substances that act non-pharmacologically, rather than inconsistently with its behavior, inappropriately trying to focus on its molecular components and reconducting its efficacy to the identification of an API acting pharmacologically. Poliprotect_(F)_ simultaneously exerts various actions, such as locally neutralizing the acid environment on the mucosa. In clinical practice, a reduction in acidity is commonly recognized as an increase in pH to above 4 [42]. This phenomenon can be considered part of Poliprotect_(F)_’s mechanism of action, as it creates a protective barrier on the gastroesophageal mucosa. Poliprotect regulates the inflammatory and antioxidant status, strengthening the mucosal barrier functionality to restore the gastroesophageal mucosa’s homeostasis and thereby recapitulating the physiological defense mechanisms that govern the gastroesophageal mucosa’s homeostasis through a 100% natural composition, rather than instructing the biological system towards non-orchestrated, isolated behaviors that, even if relevant to the onset of a therapeutic outcome at the symptom level, cannot, by default, act etiologically (Figure 9). A defect in these defense mechanisms, which could either precede or be induced via the contact of gastroesophageal refluxate with the mucosal epithelium, elicits a chain of events that can result in epithelial barrier disruption, the activation of afferent nociceptive nerves, inflammation, and oxidative stress leading to the destabilization of junctional complexes, reduced blood flow, impaired cell renewal, and reduced mucus and prostaglandin (PG) secretion [1,2,3,4,5,51]. In animal models, exposing the esophageal mucosa to acid has been shown to lead to an increase in epithelial permeability, as evidenced by epithelial dilated intercellular spaces (DISs) and TEER [52,53]. It should be noted that not only are DISs a recognized feature of experimental acid injury in animals, but also, this same abnormality has been described as a morphological feature in GERD, both non-erosive and erosive, suggesting that it is an early lesion in GERD and a clinically relevant defect in NERD [52]. As already mentioned, the current pathophysiological hypothesis of FD acknowledges the evidence of heterogeneous mechanisms behind symptoms’ generation, including factors other than acid [14]. Accordingly, in FD patients, there is sound evidence of the efficacy of treatments devoid of any ability to modify the gastric pH [54], and impaired gastric mucosal integrity has been identified via confocal endomicroscopy in FD patients as compared to healthy controls [55]. The results of electrophysiological studies highlight the capability of Poliprotect to preserve the integrity of the gastric and esophageal mucosa exposed to acidic pH. The protective activity of Poliprotect against two other harmful non-acid constituents of refluxate, i.e., bile and pepsin, was not evaluated in this study. Nevertheless, this limitation was partially addressed by a recent study demonstrating the topical protection provided via Poliprotect on Caco-2 cells and human esophageal biopsies exposed to bile acid [56]. The aforementioned in vivo evidence also further confirms Poliprotect’s efficacy against non-acid stimuli present in the explored models. Ethanol and indomethacin are the most common aggressive factors that cause damage to the gastric mucosa, with different mechanisms [57,58]. Ethanol is considered one of the agents that induce more intense gastric lesions due to a direct toxic effect on the gastric epithelium, which leads to the formation of characteristic necrotic lesions and a consequent reduction in mucus production and bicarbonate secretion. Indomethacin induces gastric lesions via a different mechanism compared to those induced via ethanol. Indomethacin is known to decrease the resistance of the gastric mucosa through the inhibition of the synthesis of prostaglandins [59]. Prostaglandins play an important protective role in the stomach because they stimulate the secretion of mucus and bicarbonate, maintain the blood flow of the mucosa, and are involved in the regulation of mucosal cell renewal [6]. Therefore, the suppression of the synthesis of prostaglandins via NSAIDs causes increased susceptibility to lesions in the gastric mucosa [57]. Poliprotect significantly decreased ethanol-induced and indomethacin-induced gastric mucosa lesions. When such observations are compiled with those concerning the localization of buffering activity, it is possible to observe that Poliprotect_(F)_’s performance indeed relies on the presence of buffering capabilities, but such a contribution can only be explained by the fact that it is exerted locally on the mucosa and not in the stomach lumen. Thus, the device establishes a multipronged interaction with the target mucosa, concomitantly mediating a number of outcomes, which together establish its therapeutic activity, delivered as a single entity acquiring emerging properties typical of the final complex matrix. Consistent with this, the protective effect upon gastric mucosa pre-treated with Poliprotect devoid of either minerals or plant material was found to be less than that of complete Poliprotect_(F)_, signifying the emergence of properties specific to the final device, based on the interactions between plant material and salts.

The evidence presented here intriguingly confirms the biological plausibility of its proven clinical efficacy in the relief of non-erosive heartburn and FD symptoms, and it seems to suggest the plausibility of the clinical efficacy of Poliprotect_(F)_ in erosive reflux disease as well. Multiple validated surveys also provide further evidence of its safety and effectiveness [60]. Poliprotect_(F)_ is 100% natural and made of edible raw materials; therefore, its contact and processing via the body could be considered inherently safe. Nevertheless, in compliance with the relevant legislation under which it is made available to patients, Poliprotect_(F)_ was submitted to the necessary characterization according to the ISO 10993 series, returning results that further strengthen its safety profile by certifying it as non-cytotoxic, non-irritating, non-sensitizing, and non-toxic via oral intake.

As a 100% natural, biodegradable, and environmentally friendly [61] medical device, Poliprotect, therefore, offers a valid alternative treatment for managing the painful clinical subtype of heartburn and FD, at least in the absence of esophageal mucosal lesions upon endoscopy.

## 4. Materials and Methods

### 4.1. Poliprotect Composition and Preparation

The composition and preparation of Poliprotect_(F)_ (NeoBianacid, Aboca S.p.A, Località Aboca 20, Sansepolcro, 52037, AR, Italy) are reported in patent nr. 102022000018165 [62]. Given the complexity of its formulation, Poliprotect’s preparation was systematically verified using a combination of chemical, physical, and biological assays including the surrogate of adhesion and barrier formation measurements hereby reported. The product was tested at a concentration defined according to its posology and the anatomical area on which the product is designed to rely in order to achieve its efficacy. Whenever relevant, in order to provide evidence that the activity of Poliprotect_(F)_ depends not on a pharmacological activity exerted upon its systemic absorption but on its biophysical properties, it was tested at a concentration mimicking the worst-case scenario, in which 100% of the formulation is systemically absorbed.

Two partial formulations were also tested in relevant experimental models: Poliprotect_(F)_ without (w/o) salt and Poliprotect_(F)_ without (w/o) plant material.

### 4.2. Cells and Cell Cultures

Human primary fibroblasts (HuDe cells) (provided by the Istituto Zooprofilattico Sperimentale della Lombardia e dell’Emilia Romagna, Via Bianchi 9, Brescia, 25124 Italy) were cultured in Minimal Essential Medium (MEM) supplemented with 10% fetal bovine serum (FBS), sodium pyruvate, streptomycin (100 μg/mL), and penicillin (100 U/mL) (all reagents from Life Technologies, Carlsbad, California, United States). The human gastric epithelial cells NCI-N87 (ATCC-CRL-5822) and the human monocytes cell line U937 (ATCC-CRL-1593.2) were maintained in RPMI-1640 (Life Technologies) supplemented with 10% FBS and the other additives indicated above, not including sodium pyruvate.

### 4.3. Assessment of Bioadhesive Properties of Poliprotect_(F)_

The human gastric epithelial cell line NCI-N87, comprising mucus-secreting cells that are histologically relevant to the site of action of the product, was used to evaluate the bioadhesive properties of Poliprotect_(F)_. Numerous methods have been proposed and are used to evaluate the mucoadhesive properties of different formulations [63,64,65,66]. The inclined plane method is based on previous work reported in specific references in the literature, and it measures mucoadhesiveness as a function of the retention of the mucoadhesive formulation in contact with a substrate. Cells were cultivated on standardized plastic substrates (Nunc Lab-Tek, Flask on Slide, Thermo Scientific, Carlsbad, California, United States) and treated with HBSS (Sigma-Aldrich, St. Louis, MO, USA) acidified to pH 1 with HCl for 30 min at 37 °C with shaking. Biological supports were washed with the complete medium in order to remove cell debris and weighed (P(s)). Then, 170 µL of Poliprotect_(F)_ solution (310 mg/mL) in simulated salivary fluid (SSF), prepared as previously described [67], was distributed on biological substrates to constitute a uniform layer. The weight of the product actually distributed P(d), and the weight of the slide loaded with the solution containing the product P(d + s), was measured. The biological substrates were then placed at an angle of 45° in a controlled atmosphere (37 °C, 5% CO_2_) for 30 s. The product was left free to slide off the supports, and their weight was measured P(a + s): upon the subtraction of the initial weight of the slide loaded with the product (P(d + s)), it was possible to calculate the weight of the product that had left the slide. By subtracting the weight of the product that had left the slide from the weight of the product actually distributed on it initially, it was possible to calculate the amount of product that remained adherent to the slide (P(a)). Next, the biological substrates, again placed at a 45° angle in a controlled atmosphere, were washed with the aid of a peristaltic pump (Ismatec Reglo ICC Digital Pump, Fisher Scientific, Hampton, New Hampshire, United States), with SSF at a constant flow rate of 0.25 mL/minute for 120 min. Every thirty minutes, the biological substrates were again weighed. The bioadhesion of Poliprotect_(F)_ to gastric cells that were not treated with an acid solution was also considered. The % bioadhesive normalized parameter was calculated as follows:

$$\%\;bioadhesion=\left(\frac{P\left(a\right)}{P\left(d\right)}\right)\times 100\;$$

In the “simplified or abiotic adhesion test”, 60 µL droplets of Poliprotect and saline solution were applied to the top of the Plexiglas surface. The system was then inclined at 45° for 30 s. After this period, the system was returned to a 0° position, and the distance traveled by the sample and the saline solution was measured in millimeters using a ruler. Six droplets per condition were considered in each of six separate experimental sessions.

### 4.4. Barrier Properties of Poliprotect_(F)_

A barrier test (BT) was performed to verify the capacity of Poliprotect_(F)_ to display barrier properties in relation to a known inflammatory agent (LPS), the major component of the outer membrane of Gram-negative bacteria and, therefore, a molecule exogenous to the human body, which triggers a detectable inflammatory reaction in cells with which it comes into contact [68]. In order to rule out any meaningful interference with the synthesis of cytokines contributed via a potentially diffusing portion of Poliprotect_(F)_ within the receiving chamber, an internal control set-up (IC) was performed at the same time as the BT. This made it possible to ensure that the barrier set-up on the inserts was prepared properly and that the sample was exerting a mechanical effect of isolating the cells from LPS. Our model used HuDe cells, responsive cells that are found throughout the skin and mucous membranes. The test requires the preparation of two chambers physically separated by a semi-permeable membrane (transwell, 6.5 mm 24-well, 0.4 µm, Fisher Scientific), which allows for the passage of sufficiently small solutes. Cells are cultured in the bottom of the lower chamber, represented by the well of the cell culture plate. Poliprotect_(F)_ was dissolved in 0.9% saline solution to obtain a final concentration of 260 mg/mL. Following this, 70 μL of the sample solution was layered on the inner surface of the transwell membrane separating the two chambers in order to evaluate its barrier effect against the free passage of the inflammatory agent LPS (1 μg/mL), which was added to the bottom of the upper chamber for 1 h, as represented by the cell culture insert. Depending on the barrier-constituting capacities of the sample, a decrease in the migration of the inflammatory agent from the upper chamber will be observed, resulting in less stimulation of the cells towards the production of cytokines. The test also included a positive control in which cells were treated with LPS in the absence of the tested sample, along with a negative control (untreated cells) in which the cells were treated with the culture medium alone, in the absence of the sample being tested. The extent of the inflammatory reaction was estimated after 24 h via a quantitative determination of IL-6 release in the lower chamber—a response typical of the late phase of the inflammatory reaction [69]. The IC involves the same steps as the BT, but with different dynamics, apt to highlight any inflammatory activity exerted via mediators putatively diffusing from the product barrier; the same quantity of LPS used in the BT was left free to diffuse from the upper to the lower chamber and, after just 1 h, 70 μL of the sample solution was layered on the inner surface of the transwell membrane. In this set-up, the observation of anti-inflammatory activity could be ascribable to the diffusion of interactors from the barrier, rather than to the barrier effect itself. The quantification of IL-6 in supernatants was performed using ELISA (ab178013, Abcam, Cambridge, United Kingdom), according to the manufacturer’s instructions. Following the collection of the supernatants, cell viability was assessed via an MTT assay (Sigma-Aldrich), according to the manufacturer’s instructions. To confirm the physical/mechanical nature of the observed phenomena, an ad hoc assay conducted in abiotic conditions was also performed. This assay was based on the principle that reducing the passage of the molecule dextran–Rhodamine B (70,000 molecular weight, D1841, Invitrogen, Carlsbad, California, United States) after the application of the product is considered an index of the protective capability of the formulation compared to an untreated control (no formulation application, positive control). The test requires the preparation of two chambers physically separated by a semi-permeable membrane (HTS Transwell-96-3391, Corning, New York, NY, USA). Poliprotect_(F)_ was allowed to sit on the surface of the transwell for 10 min prior to the application of dextran–Rhodamine B. The receptor solution was then collected after 1 h and was read using a Varioskan™ reader (Thermo Scientific) at 570 excitation and 590 emission, and it was considered that the barrier effect could be determined by measuring the fluorescence of the receiver-plate well solution. After the quantity of dextran–Rhodamine B present in the different experimental conditions was calculated, the percentage of the barrier effect was calculated as follows:

$$\%\;barrier\;effect=100-\left(\frac{sample\;dextran\;concentration}{mean\;dextran\;concentration\;of\;ctrl+}\right)\times 100$$

### 4.5. Radical Scavenging Activity

To measure radical scavenging activity, a cell-based model widely used in the literature was performed [70,71]. HuDe cells were treated with AAPH (Sigma-Aldrich), which induces exogenous pro-oxidant damage and, thus, the production of endogenous ROS. For the assay, HuDe cells were seeded in 96-well plates at 30,000 cells/well in Antioxidant Treatment Medium (ATM, 10 mM HEPES, Sigma-Aldrich). The ATM medium was removed, the cells were washed, and the fluorescent probe 6-carboxy-2′,7-dichlorodihydrofluorescein diacetate (60 μM H2DCDFA, Life Technologies) was added to the ATM medium as an indicator of the presence of ROS in cells. The cells were then placed in an incubator for 20 min, the solution was removed, and treatment solutions (Poliprotect_(F)_ 750 μg/mL + AAPH 500 μM; ascorbic acid 284 μM Sigma-Aldrich + AAPH 500 μM; AAPH 500 μM) prepared in Oxidant Treatment Medium (OTM:HBSS 1 mM, 10 mM HEPES; Life Technologies) were added to the cells. The fluorescence emitted by H2DCDFA was measured every 10 min for a total of 100 min with a Varioskan™ reader (Thermo Scientific) and quantitatively correlated with the production of free radicals in the cells. Fluorescence values were normalized with respect to the cell numbers determined by nuclear mask staining (Life Technologies).

### 4.6. Buffering Activity and Its Localization

Acid secretion is an important physiological process of the stomach, both for the activation of pepsinogen to initiate the digestive process and for killing bacteria or inhibiting their growth [72]. Evidence of the presence of a surface pH gradient on top of the gastric mucosa can be found in several studies [73]. The excessive secretion of gastric acid and pepsinogen damages the gastric mucosa and increases mucosal permeability, leading to dyspeptic symptoms and heartburn with substernal pain or burning sensation. These are critical factors for the development of gastroesophageal diseases [2,74]. To determine the buffering activity of Poliprotect_(F)_, two studies were performed. In the first study, we assessed its ability to buffer an acidic solution at pH values typical of gastric levels during digestion. Poliprotect_(F)_ was tested at a concentration of 310 mg/mL in water, and pH paper was used for pH measurements (scale of 0–14) as follows:

(a) One hundred μL of a solution of HBSS acidified to pH 1 with HCl was distributed on pH paper to constitute a uniform layer, and the color change was read by comparing the color of the strip with the reference scale.
(b) One hundred μL of a solution of Poliprotect_(F)_ was distributed on pH paper to constitute a uniform layer, and the color change was read by comparing the color of the strip with the reference scale. An additional 100 µL of HBSS pH 1 was layered on top of the thus-formed barrier, and the color change was read as described above.
(c) One hundred μL of a basic solution of HBSS pH 8 was distributed on pH paper to constitute a uniform layer, and the color change was read by comparing the color of the strip with the reference scale. An additional 100 µL of HBSS pH 1 was layered on top of this solution, and the color change was read as described above.

The second study was performed to determine whether the buffering activity of Poliprotect_(F)_ is therapeutically relevant in terms of localization at the local level on the mucosa or in the stomach lumen. To do so, we studied Poliprotect_(F)_’s ability to buffer in a test tube an acid solution at pH values typical of the gastric levels during digestion [42,43], across a range of concentrations that would signify either a local or luminal mechanism of action. This performance was compared to that of a commercial antacid (magnesium hydroxide 400 mg + aluminum oxide 400 mg, hydrate) known to act in the lumen of the stomach. Two concentrations per product were considered for pH measurement. Poliprotect_(F)_ was tested at concentrations of 310 mg/mL and 3.4 mg/mL. The commercial antacid was tested at concentrations of 640 mg/mL and 7 mg/mL. The first concentration was calculated considering both the maximum single recommended dose (one tablet equal to 1.55 g for Poliprotect_(F)_ and two tablets equal to 3.2 g for the commercial antacid) and the salivary activity during product application (total volume of 5 mL) [75]. The second was calculated considering both the maximum single recommended dose and the total gastric volume at maximum satiety (456 mL) [76]. The products were dissolved in water acidified at pH 1.5 by adding HCl (Sigma-Aldrich) to mimic the gastric levels during digestion [43]. pH recording was started immediately after vigorous mixing using an Orion Star A211 pH meter (Thermo Scientific). Three biological replicates were considered. An untreated sample was considered in all pH measurements.

### 4.7. Effects of Treatment with Poliprotect_(F)_ on Human Gastric Epithelial Cells

In studies conducted in accordance with the principles of systems biology, we evaluated the effects of Poliprotect_(F)_’s barrier formation on human cells relevant to its intended use, highlighting the emergence of complex responses from the biological system, and providing details concerning the modalities through which they were achieved.

#### 4.7.1. Cell Treatments and Transepithelial Electrical Resistance (TEER) Measurement

Human gastric epithelial cells (NCI-N87) were seeded on 24-well plate polyester membrane inserts (Corning ^TM^ 3470) and treated 8 days post-seeding. A schematic representation of the experimental design can be found in Supplementary Figure S2. On the day of the treatment, the cells were first pre-incubated for 1 h in an incubator, with shaking (80 rpm), in HBSS pH 7.4. In this phase, HBSS was provided to both the apical and basolateral sides. The basolateral HBSS was then replaced with 600 µL of fresh HBSS pH 7.4, and the apical HBSS was then replaced with treatment solutions as follows:

(a) Acid-induced damage: The apical HBSS was replaced with 26.8 µL of HBSS acidified to pH 1 with HCl, and incubation continued with shaking for 30 min.
(b) Poliprotect_(F)_: The apical HBSS was removed, and cells were treated with 3.2 µL of the product dissolved in HBSS pH 1 at a 310 mg/mL concentration. An additional 26.8 µL of HBSS pH 1 was layered on top of the thus-formed barrier to represent the presence of the acidic stress. The incubation continued with shaking for 30 min.
(c) Control: The apical HBSS was replaced with 26.8 µL of fresh HBSS pH 7.4, and incubation continued with shaking for 30 min.

To explore whether Poliprotect_(F)_’s activity depends on systemic absorption, we verified its inability to counter acid-induced damage or its consequences when dissolved at a concentration mimicking the worst-case scenario, in which 100% of the product is systemically absorbed. After pre-incubation for 1 h in an incubator (37 °C, 5% CO_2_), with shaking (80 rpm), in HBSS pH 7.4, the treatments were performed as follows:

(a) Acid-induced damage: The apical and basolateral HBSS pH 7.4 was replaced with HBSS pH 1 (26.8 µL) and RPMI-1640 culture medium (600 µL), respectively. Incubation continued with shaking for 30 min.
(b) Poliprotect_(F)_: The apical and basolateral HBSS pH 7.4 was replaced with HBSS pH 1 (26.8 µL) and Poliprotect_(F)_ solution (600 µL) at a concentration of 110 µg/mL in RPMI-1640, respectively. The Poliprotect_(F)_ solution was prepared by diluting the solution used for the apical treatment in RPMI-1640 (310 mg/mL), and incubation continued with shaking for 30 min.
(c) Control: The apical HBSS 7.4 was replaced with 26.8 µL of fresh HBSS pH 7.4, the basolateral HBSS 7.4 was replaced with 600 µL of RPMI-1640, and incubation continued with shaking for 30 min.

At the end of the incubation period, the treatment solutions were removed, and the apical and basolateral sides were washed with HBSS pH 7.4. Fresh medium was then added to both the apical and basolateral sides. The plates were incubated for 30 min at 37 °C. Both untreated cell monolayers and transwells with the filter insert without cells were used as controls. TEER was applied to measure the barrier integrity by placing the appropriate electrodes in the apical and basolateral positions according to the manufacturer’s instructions (EVOM^2^, World Precision Instrument, Friedberg, Germany). Raw values were corrected by subtracting the intrinsic electrical resistance (Ω) of the filter and were multiplied by the insert surface area (cm^2^). Cell viability was evaluated using the ToxiLightTM BioAssay Kit (LT07-217, Lonza, Basel, Switzerland) according to the manufacturer’s instructions.

#### 4.7.2. Gene Expression and Bioinformatics Analysis

At the end of the incubation period, the cells were washed, lysed, and collected in RLT buffer (Qiagen, Hilden, Germany) supplemented with β-mercaptoethanol (Sigma-Aldrich) and DX reagent (Qiagen) for gene expression analysis experiments. The total RNA was extracted from the cell lysates using a QIAsymphony RNA Kit with the QIAsymphony SP instrument (Qiagen). The quality and quantity of RNA was determined via A230, A260, A280, and A320 measurements on a Varioskan™ device. The integrity of RNA was checked using a 2100 expert_Eukaryote Total RNA Nano Kit (Agilent, Santa Clara, CA, USA). Whole-transcriptome expression profiles were evaluated using a Human Clariom™ S Pico Assay HT on a GeneTitan MC Instrument (Applied Biosystems, Waltham, MA, USA), following the manufacturer’s instructions. Data analysis was performed using the Transcriptome Analysis Console (TAC version 4.0.2.15, Thermo Scientific) software, which provides a list of differentially expressed genes based on the limma package approach (Bioconductor), using a threshold of *p*-value ≤ 0.05 [77]. The Ingenuity Pathway Analysis, version 84978992, software (IPA, Qiagen) was then used to extract biological meaningfulness from the “high-throughput” gene expression data. To capture extremely early, potentially both canonical and transition-state events in the context of acid-induced damage, and to grasp the biological meaningfulness of events triggered at the 30 min timepoint, we monitored several “Canonical pathways” (CPs) and “Diseases and Biofunctions” (DBs) obtained from the IPA core analysis (with z-score cutoffs of ≤−1.7 and ≥+1.7) of genes identified as differentially expressed in the samples subjected to apical treatments. Among the significant CPs and DBs that emerged from the analysis, we focused on those involving anti-inflammatory activity and the stimulation of tissue self-healing abilities, which we deemed particularly relevant in the present setting. The lists of differentially expressed genes were studied in the context of said CPs and DBs and, by means of the “IPA Molecule Activity Predictor” (MAP) tool, up- or downmodulations were determined. The same analysis was conducted on samples subjected to basolateral treatments in order to ensure that any observed activity did not depend on systemic absorption. In order to further elaborate on the results thus generated, gene expression profiles (*p*-value ≤ 0.05) were investigated using the “IPA Path Analysis” tool. The MAP tool was used in order to explore the impact of cell treatments on additional biofunctions identified using the “IPA Bioprofiler” tool and associated with the following categories: organismal injury; tissue morphology; cellular assembly and organization; gastrointestinal diseases; differentiation and proliferation of epithelial and stromal cells; healing and inflammation.

To define the numerical z-score of each of the biofunctions identified via “IPA Path Analysis”, the network was integrated with BFs, with z-scores attributed via the core analysis. The image was downloaded as a JPEG file and subjected to a basic intensity quantification protocol using ImageJ software, v1.53e. In brief terms, the image was converted into an 8-bit format, and every mean of BF intensity was calculated using the “measure” tool (Supplementary Figure S4). The obtained intensity values of BFs derived from the core analysis were used to create a sigmoidal 4PL curve, which we used to interpolate values of BFs derived from “IPA Path analysis”, thus obtaining inferred values of the corresponding manual BFs. All values were displayed as heatmaps. Curve construction and the interpolation of data were performed using GraphPad Prism version 9.3.0.

### 4.8. Preservation of the Permeability of Gastric and Esophageal Mucosae

The Ussing chamber provides a valuable method for assessing the permeability of the murine gastric and esophageal mucosae [78,79]. Two experiments were performed, focusing on outcomes at the stomach and esophagus levels, respectively, each considering 10 ICR (CD-1) adult male mice (38–40 g each, Harlan Laboratories srl, Udine, Italy) divided into 2 groups (5 animals/group). Throughout the experiment, all mice were kept in temperature- and humidity-controlled conditions with a 12 h light/dark cycle. Food and water were available ad libitum. All mice selected for the study were identified via a numbered plastic mark applied to the right ear and were randomly assigned to the different groups at the beginning of the study. Cages were identified with a tag. The study was performed in accordance with the directives and the ethical rules of the European Community’s Council for the Care and Use of Laboratory Animals (86/609/EEC).

One group received 100 μL of saline solution via oral gavage. The second group received 100 μL of Poliprotect_(F)_ (650 mg/kg). Thirty minutes after oral treatment, all animals were anesthetized and prepared for stomach or esophagus explant before being placed in preheated and oxygenated Krebs buffer (pH 7.35). The organs were then dissected, and a fragment of ~0.25 cm^2^ was excised. In order to measure gastric/esophageal permeability, excised fragments were mounted between the two emi-chambers of an Ussing system (0.125 cm^2^ opening). Two calomel voltage-sensitive electrodes and two Ag-AgCl current-passing electrodes (EVC-4000, World Precision Instrument) were connected to the Ussing chamber via agar bridges. The assembled chamber was placed in a block connected to a circulating warm water bath. Then, 5.5 mM D-glucose (Sigma-Aldrich) was added to the mucosal and serosal sides of the chamber in order to provide an energy source and an equivalent osmolality between the two sides. Once the gastric/esophageal membrane was mounted in the Ussing chamber, the system was allowed to stabilize for 10 min. After that period, a difference in electrical potential was applied between the two sides of the membrane, and the transepithelial electrical potential difference (mV) across the mucosal membrane and the short-circuit current (μAmp) were measured directly for 15 min, while the transmembrane resistance was calculated as ohm x cm^2^. Then, to induce damage to the mucosa, the buffer solution was replaced with an acid solution at pH 3, and after 10 min, the resistance was measured again.

### 4.9. Lack of Direct Anti-Inflammatory Activity in a Human Macrophage Model (U937)

To provide further evidence that the anti-inflammatory activity of Poliprotect_(F)_ depends on its ability to form a barrier locally in the GI tract and not on any activity exerted directly on immune cells as a consequence of its absorption, the inability of Poliprotect_(F)_ to counter inflammation evoked via LPS in a U937 cell model was verified at a concentration mimicking the worst-case scenario, in which 100% of the device is systemically distributed. A cell-based model used in the literature was used [80,81]. U937 cells were differentiated into macrophages upon treatment with 10 ng/mL PMA (phorbol 12-myristate 13-acetate, Sigma-Aldrich) for 48 h in RPMI + 10% FBS. At the end of the differentiation period, the culture medium was replaced with RPMI + 10% FBS for 24 h. Cells were then harvested and resuspended in RPMI + 1% FBS, seeded in 48-well plates (3 × 10^5^ cells/well in 300 µL), and left to adhere for 2.5 h. The cells were then either left untreated or treated with Poliprotect_(F)_ (111 µg/mL) or 100 nM of the anti-inflammatory drug dexamethasone (DEX, Sigma-Aldrich). After 2 h, 1 µg/mL LPS was added. After an incubation period of 24 h, the culture supernatants were analyzed with the Bio-PlexProTM Human Cytokine 27-Plex assay kit (Bio-Rad, Hercules, CA, USA ) to assess the effects of the tested samples on the secretion of 27 cytokines and chemokines, according to the manufacturer’s instructions. The cell viability at the end of the treatment period was assessed via an MTT assay, according to the manufacturer’s instructions. The variation in cytokine secretion values (fold-change) observed in response to different treatment conditions was calculated by normalizing every value against the “LPS” sample. IPA was then used to interpret the biological functions associated with the resulting dataset. The “Core Analysis” function was used to analyze data from individual treatment conditions. A cutoff value of 1.5 was applied to the uploaded fold-changes in order to signify biological relevance. The “Comparison Analysis” function was then used to compare results obtained for the different treatment conditions considered. Only z-scores greater than 2 and lower than −2 were considered meaningful. Only highly correlated biofunctions resulting from the analyses were exported for heatmap representation purposes.

### 4.10. Protective Activity of Poliprotect_(F)_ in an Animal Model of Erosive Gastropathy

#### Animal Models of Erosive Gastropathy

Two models of erosive gastropathy were used in these experiments: an ethanol-based model and an indomethacin-based model. Ethanol and indomethacin are the most common aggressive factors that cause damage to the gastric mucosa via different mechanisms [57,58]. Therefore, these models can be considered reliable tools for evaluating the protective activity of the product. For this purpose, male Wistar rats (225–250 g, Harlan) were used. Throughout the experiment, all rats were kept in individual cages in temperature- and humidity-controlled conditions, with a 12 h light/dark cycle. Food and water were available ad libitum.

For each experiment, the animals fasted for 48 and 24 h for the ethanol- and indomethacin-based models, respectively, and they were divided into 4 groups. During fasting, they were kept in metal cages with grid floors to avoid coprophagic behavior during this period. This study was performed in accordance with the directives and the ethical rules of the European Community’s Council for the Care and Use of Laboratory Animals (86/609/EEC).

(a) Rats without lesions, treated with vehicle, without other administrations (10 mL/kg per os) (*n* = 6, SHAM).
(b) Rats treated with a vehicle and the subsequent administration of ethanol or indomethacin (10 mL/kg); (*n* = 12; ethanol or indomethacin).
(c) Rats treated with Poliprotect_(F)_ (650 mg/kg), Poliprotect_(F)_ without salt (472 mg/kg), or Poliprotect_(F)_ without plant components (587 mg/kg) (all per os), and the subsequent administration of ethanol or indomethacin (*n* = 12).
(d) Rats treated with a reference compound (ranitidine, 50 mg/kg) and the subsequent administration of ethanol or indomethacin (*n* = 12).

In the ethanol-based model, gastric lesions were induced 1 h after treatment with the study compounds through intragastric administration of 1 mL of ethanol. In the indomethacin-based model, gastric lesions were induced 1 h after treatment with the study compounds through an intragastric administration of 40 mg/kg indomethacin. One hour or four hours after the administration of ethanol or indomethacin, respectively, the rats were euthanized and their stomachs were immediately collected for macroscopic lesion area analysis. The length of each hemorrhagic lesion was measured, and an arbitrary value was assigned according to a standard method [82]: <1 mm: score = 1; 1–2 mm: score = 2; >2 mm: score equal to major length. The total number and magnitude of gastric lesions were expressed as an ulcerogenic index (UI).

### 4.11. Statistical Analyses

Statistical analysis was performed using GraphPad Prism Version 9.3.0. Statistical significance was tested using unpaired *t*-tests and one-way analysis of variance (ANOVA) with Dunnett’s, Tukey’s, Sidak’s, or uncorrected Fisher’s LSD post hoc tests, as appropriate. *p*-values < 0.05 were considered significant. All data are presented as the mean ± standard deviation (SD) or standard error (S.E.M).

## Data Availability

Data is contained within the article and Supplementary Materials.

## Supplementary Materials

The following supporting information can be downloaded at https://www.mdpi.com/article/10.3390/ijms26031181/s1.
