# Supplementary material for: A Non-Pharmacological Paradigm Captures the Complexity in the Mechanism of Action of Poliprotect Against Gastroesophageal Reflux Disease and Dyspepsia
Source: Int J Mol Sci. 2025 Jan 29;26(3):1181. doi: 10.3390/ijms26031181 (PMC11818618; doi:10.3390/ijms26031181)
Supplement: Supplementary file 1 [file ijms-26-01181-s001.zip › ijms-3403461-supplementary.pdf]

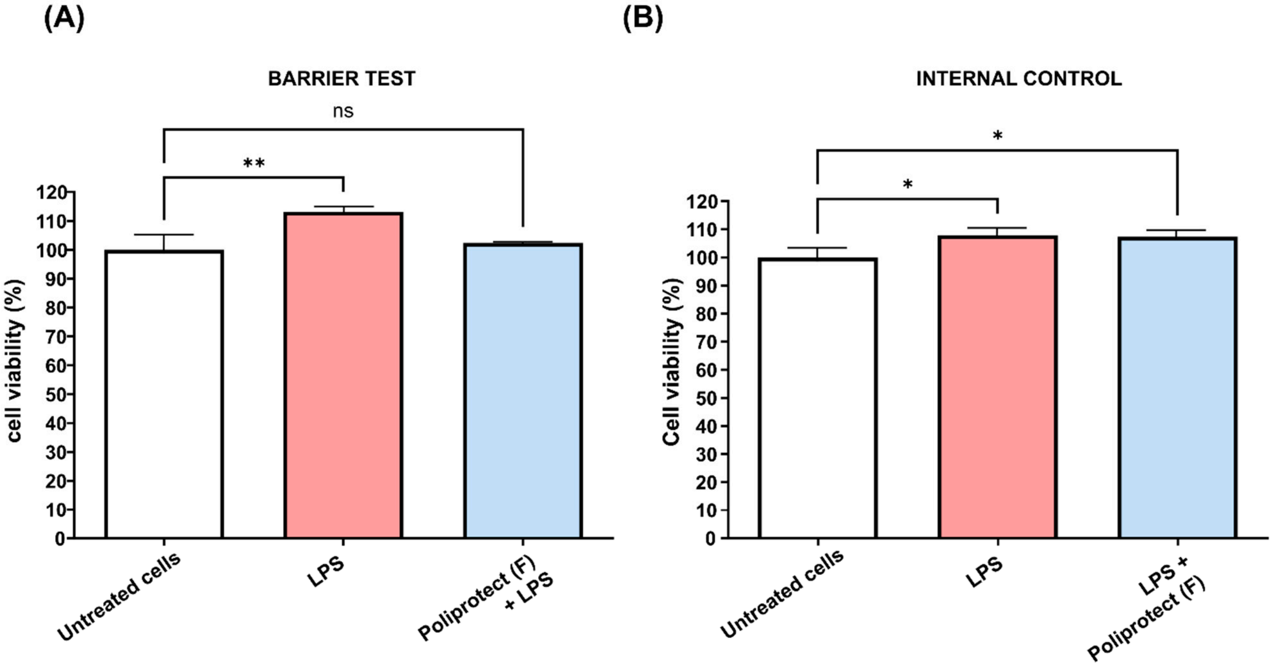

Supplementary Figure S1: Impact of treatments on cell viability in the barrier test and internal control set-up. Values are the mean ±SD: one-way ANOVA with Dunnett's post hoc test. **p* -value<0.05; ***p* -value <0.01; *ns*: not significant.

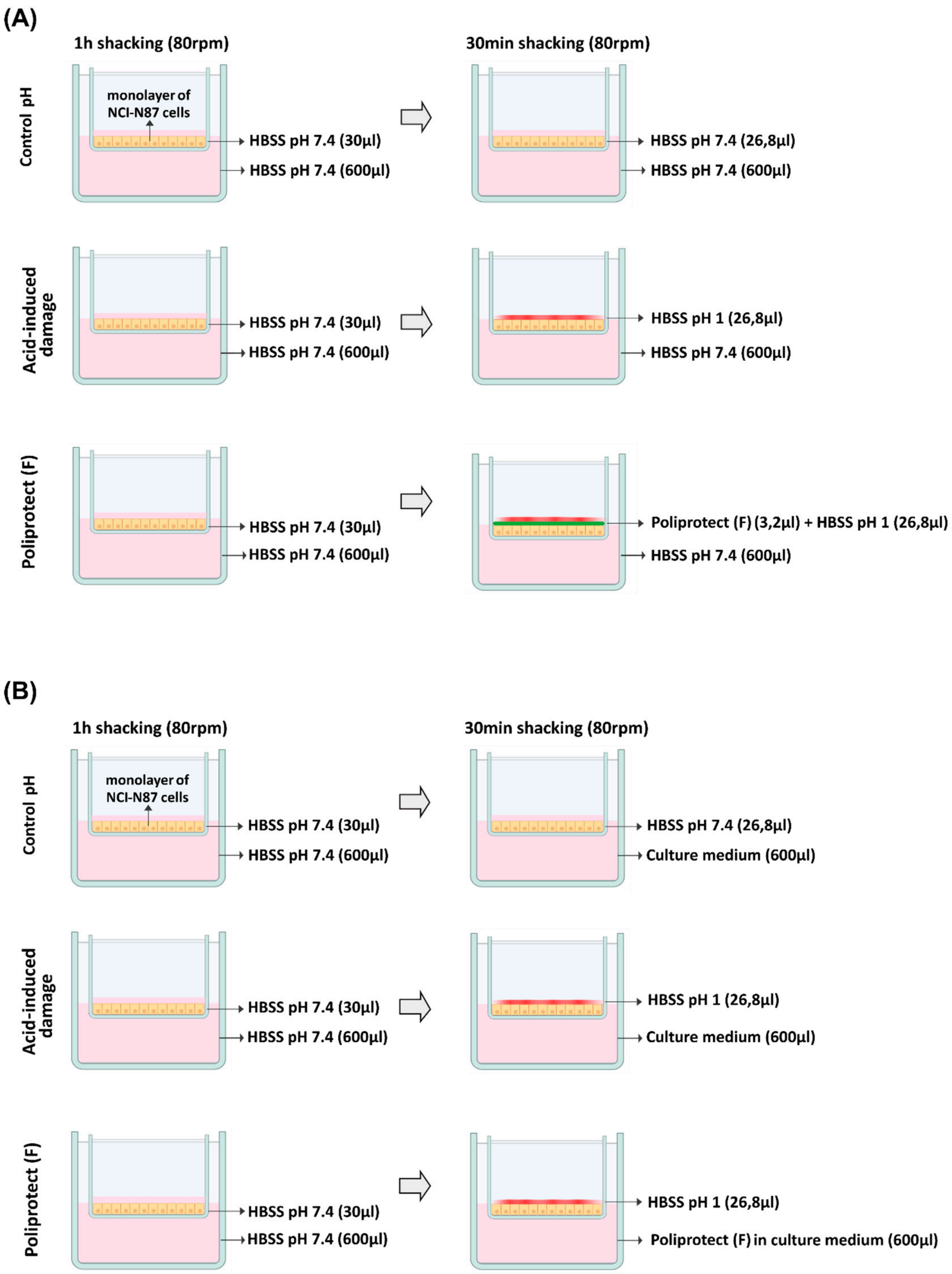

Supplementary Figure S2: Experimental design of the study of the effects of treatments with Poliprotect$_{(F)}$ on human gastric epithelial cells (NCI-N87). **(A)** Apical treatment. **(B)** Basolateral treatment.

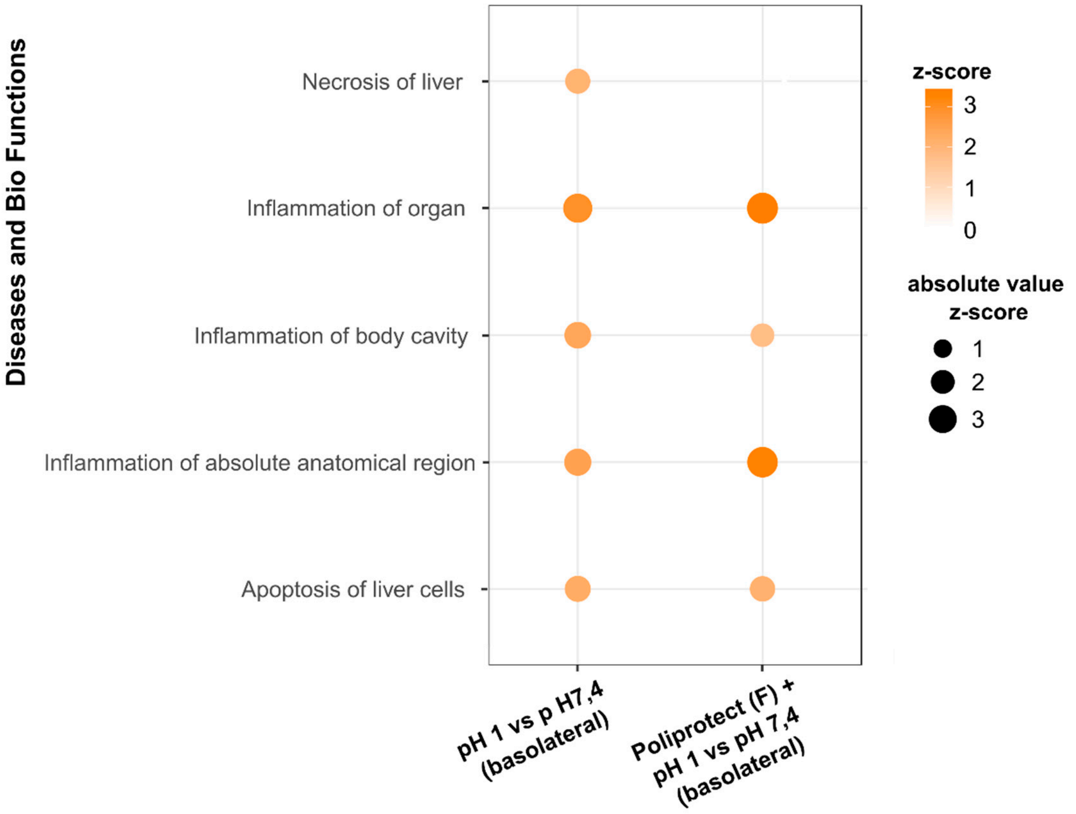

Supplementary Figure S3: Disease and biofunctions, resulting from IPA core analysis, observed when Poliprotect(F) was applied to the basolateral side (following the same parameters of the core analysis performed for apical data).

Z-scores calculation of the bio functions identified by "IPA Path Analysis"

pH 1 vs pH 7,4

(A)

Biofunctions	Pixel intensity	Reference Z-scores obtained from IPA core analysis
Sphere formation of tumor cell lines	131	-1,782
Colony formation of fibroblasts	113	-1,276
Disorder of hair	110	-1,134
Growth of hair	98	-1
Endoplasmic reticulum stress response	55	-0,509
Quantity of L-amino acid	11	-0,106

(B)

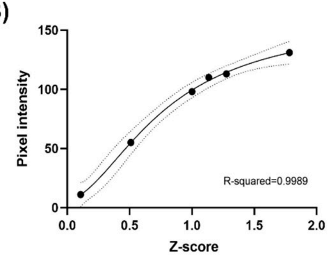

(C)

Biofunctions	Pixel intensity	Inferred Z-score
Healing of physiological and anatomical system	119	-1,387
Healing	117	-1,337
Formation of tight junctions	97	-0,964
Developmental process of tight junctions	96	-0,950
Quantity of tight junctions	96	-0,950
Barrier function of tight junctions	96	-0,950
Microtubule dynamics	56	-0,518
Organization of cytoplasm	44	-0,417
Organization of cytoskeleton	43	-0,409

(D)

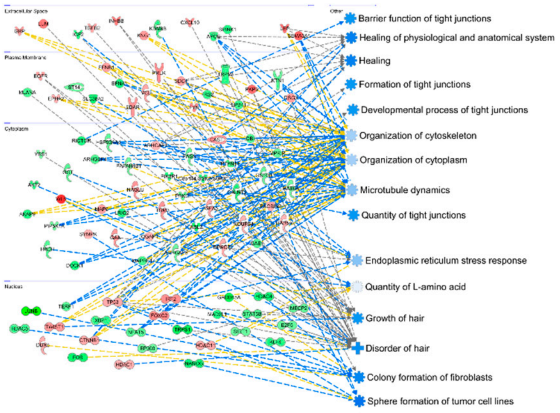

Poliprotect (F) + pH 1 vs pH 7,4

(E)

Biofunctions	Pixel intensity	Reference Z-scores obtained from IPA core analysis
Differentiation of keratinocytes	140	2,550
Cytostasis	137	2,101
Permeability of vasculature	136	1,730
Contact growth inhibition	114	1,294
Apoptosis of embryonic cell lines	102	1,062
Cell death of fibroblast cell lines	63	0,590

(F)

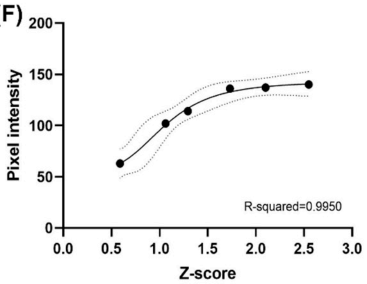

(G)

Biofunctions	Pixel intensity	Inferred Z-score
Organization of cytoplasm	135	1,853
Microtubule dynamics	133	1,742
Organization of cytoskeleton	132	1,696
Formation of tight junctions	130	1,616
Developmental process of tight junctions	129	1,581
Healing	129	1,581
Healing of physiological and anatomical system	117	1,296

(H)

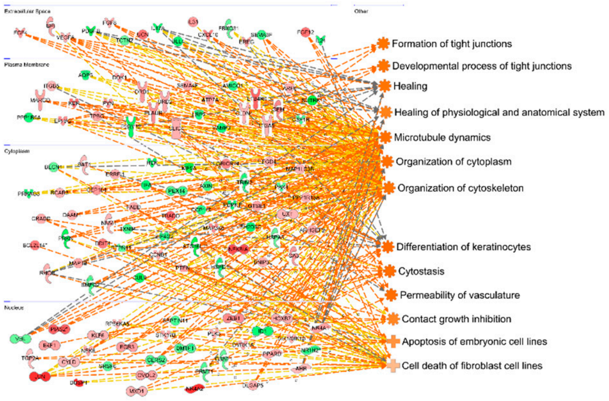

Supplementary Figure S4: Z-scores calculation of the biofunctions identified by "IPA Path Analysis". **(A,E)** Reference Z-scores obtained from the “Core analysis” and their relative values of color intensity. **(B,F)** Interpolation curves of reference Z-scores and color intensities. **(C,G)** Calculated Z-score values of each of the biofunctions identified by "IPA Path Analysis”. **(D,H)** IPA networks used to pick the biofunction color intensities.

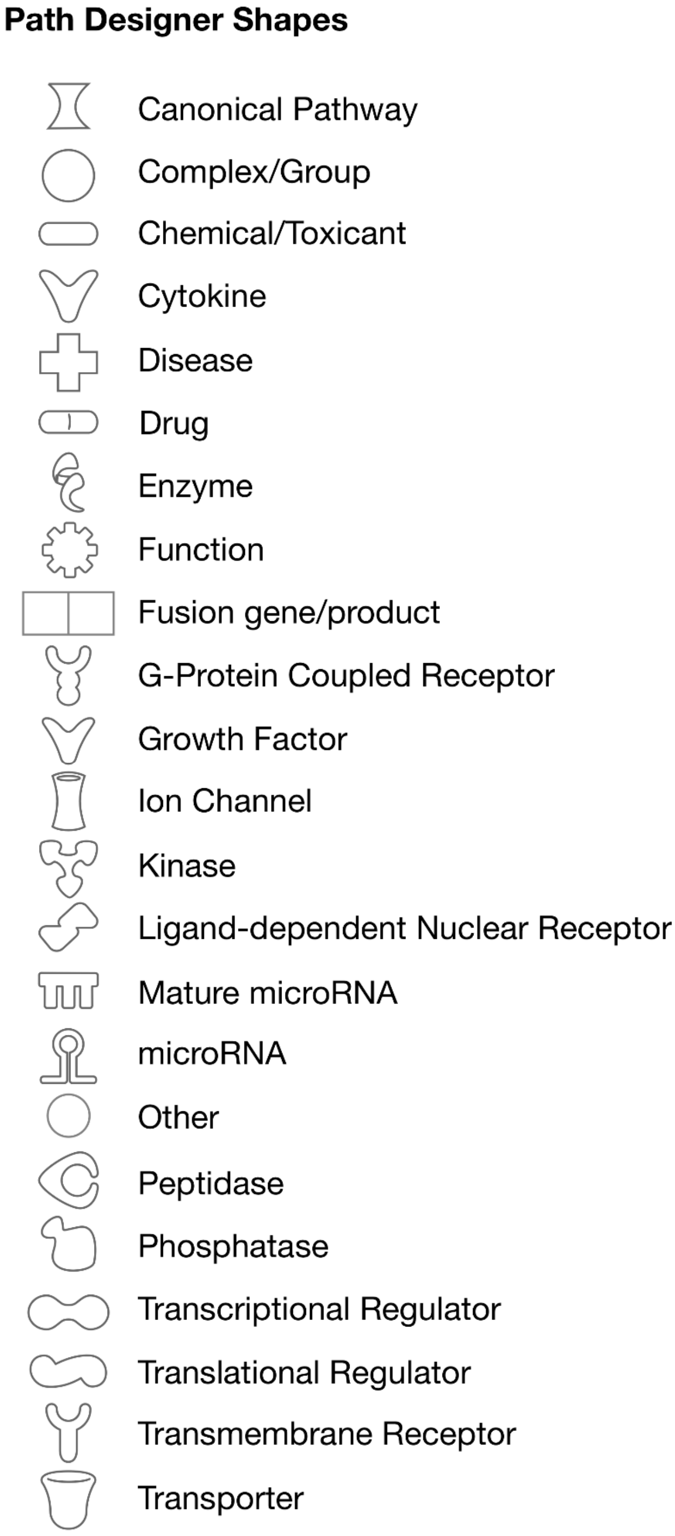

Supplementary Figure S5: Path Designer Shapes of genes and diseases and biofunctions shown in figure 5.
